# Supplementary figures and images for: Reliability of motion phase identification for long-track speed skating using inertial measurement units
Source: PeerJ. 2024 Sep 27;12:e18102. doi: 10.7717/peerj.18102 (PMC11441384; doi:10.7717/peerj.18102)

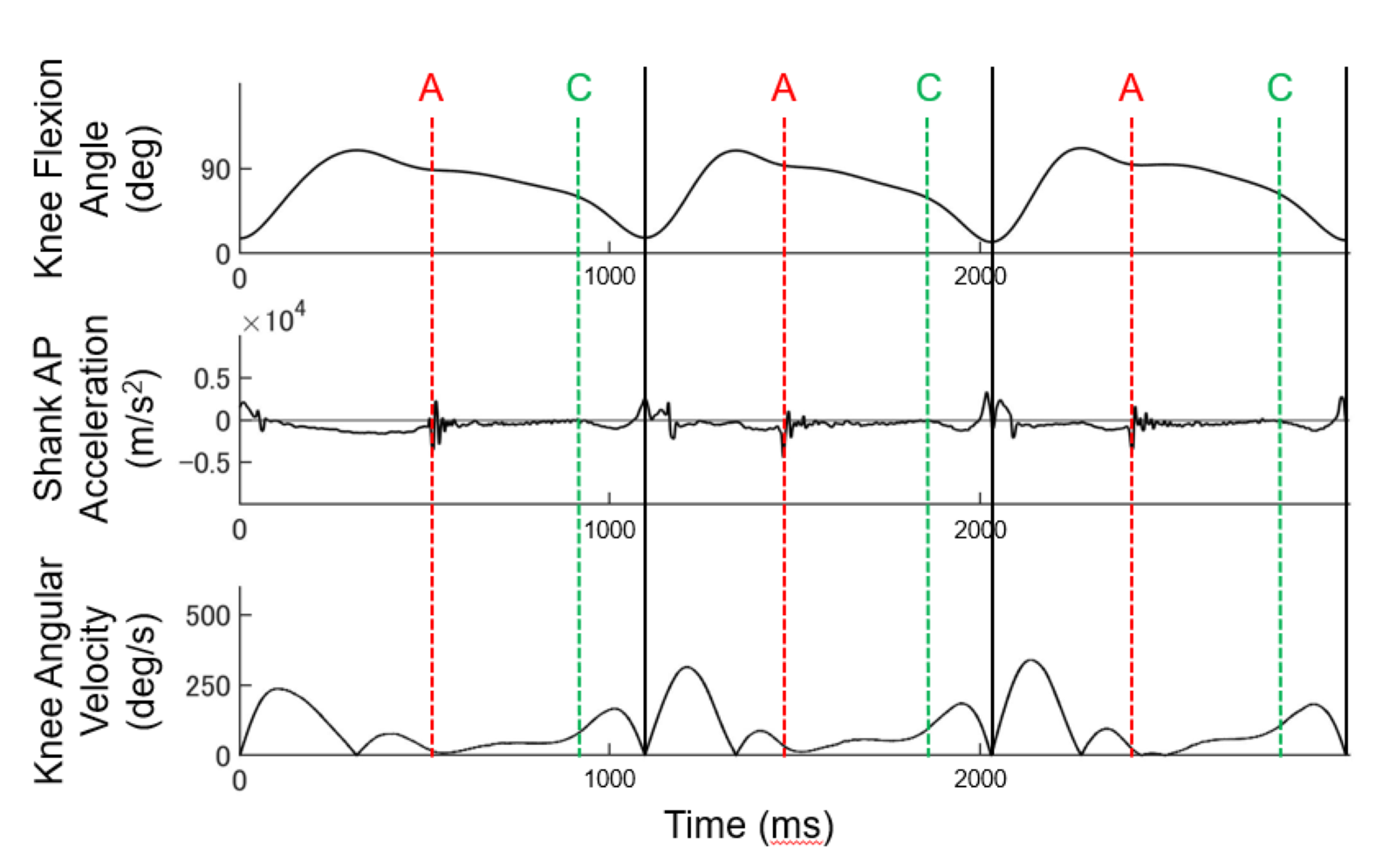

Supplement: Figure S1 — Knee flexion angle: the relative angle between the thigh and shank segments in the sagittal plane; Shank AP Acceleration: Vertical acceleration of Shank sensor; Knee Angular Velocity: Knee flexion angular velocity; Black lines: 1 stroke cycle; Red dotted lines (A): Onset; Green dotted lines (C): Push-off; note that Edge-flip is not shown since Edge-flip is absent in the curve section. [file peerj-12-18102-s001.png]
